# SARS-CoV-2 specific serological pattern in healthcare workers of an Italian COVID-19 forefront hospital

**DOI:** 10.1186/s12890-020-01237-0

**Published:** 2020-07-29

**Authors:** Giovanni Sotgiu, Alessandra Barassi, Monica Miozzo, Laura Saderi, Andrea Piana, Nicola Orfeo, Claudio Colosio, Giovanni Felisati, Matteo Davì, Alberto Giovanni Gerli, Stefano Centanni

**Affiliations:** 1 Clinical Epidemiology and Medical Statistics Unit, Department of Medical, Surgical, Experimental Sciences, University of Sassari, Sassari, Italy; 2 Clinical Epidemiology and Medical Statistics Unit, Department of Clinical and Experimental Medicine, University of Sassari, Sassari, Italy; 3 Laboratory of Clinical Chemistry, ASST Santi Paolo e Carlo, San Paolo Hospital, Department of Health Sciences, Università degli Studi di Milano, Milan, Italy; 4 Department of Pathophysiology and Transplantation, Università degli Studi di Milano, Milan, Italy; 5 Fondazione IRCCS Ca’ GrandaOspedale Maggiore Policlinico, Milan, Italy; 6 Medical Direction ASST Santi Paolo e Carlo, Milan, Italy; 7 Occupational Health Unit, International Center for Rural Health, ASST Santi Paolo e Carlo, Department of Health Sciences, Università degli Studi di Milano, Milan, Italy; 8 Head and Neck Dapartment, ASST Santi Paolo e Carlo, San Paolo Hospital, Department of Health Sciences, Università degli Studi di Milano, Milan, Italy; 9 Respiratory Unit, ASST Santi Paolo e Carlo, San Paolo Hospital, Department of Health Sciences, Università degli Studi di Milano, Milan, Italy; 10 Management Engineering Tourbillon Tech srl, Padova, Italy

**Keywords:** Seroprevalence, COVID-19, SARS-CoV-2, IG, HCWs

## Abstract

**Background:**

COVID-19 is an infectious disease caused by a novel coronavirus (SARS-CoV-2). The immunopathogenesis of the infection is currently unknown. Healthcare workers (HCWs) are at highest risk of infection and disease.

Aim of the study was to assess the sero-prevalence of SARS-CoV-2 in an Italian cohort of HCWs exposed to COVID-19 patients.

**Methods:**

A point-of-care lateral flow immunoassay (BioMedomics IgM-IgG Combined Antibody Rapid Test) was adopted to assess the prevalence of IgG and IgM against SARS-CoV-2. It was ethically approved (“Milano Area 1” Ethical Committee prot. n. 2020/ST/057).

**Results:**

A total of 202 individuals (median age 45 years; 34.7% males) were retrospectively recruited in an Italian hospital (Milan, Italy). The percentage (95% CI) of recruited individuals with IgM and IgG were 14.4% (9.6–19.2%) and 7.4% (3.8–11.0%), respectively. IgM were more frequently found in males (24.3%), and in individuals aged 20–29 (25.9%) and 60–69 (30.4%) years. No relationship was found between exposure to COVID-19 patients and IgM and IgG positivity.

**Conclusions:**

The present study did show a low prevalence of SARS-CoV-2 IgM in Italian HCWs. New studies are needed to assess the prevalence of SARS-CoV-2 antibodies in HCWs exposed to COVID-19 patients, as well the role of neutralizing antibodies.

## Background

The coronavirus SARS-CoV-2 (Severe Acute Respiratory Syndrome Coronavirus 2) is a newly emerging virus which can spread rapidly. The SARS-CoV-2-related disease 2019 (COVID-19) has been declared a public health emergency by the World Health Organization [1]. After initial epidemiological reports in China, Italy has been one of the first countries for incident cases and deaths [2, 3].

Human-to-human transmission via droplets, contaminated hands or surfaces has been described. The incubation time can range from 2 to 14 days. Early diagnosis, and supportive critical care can save lives of infected cases [4]. Real time reverse transcriptase polymerase chain reaction (RT-PCR) is the gold-standard for the virological diagnosis. However, several cases of false-negative patients have been described owing to low viral load [5] and inappropriate sample collection. The consequence can be dramatic: contagious patients can transmit viruses and hamper any public health efforts to contain the viral circulation [6]. Serological testing can indirectly detect the presence of infection. Detection of immunoglobulin (Ig) M in combination with PCR can increase the diagnostic accuracy. IgM are produced during the acute phase of the infection, followed by high-affinity IgG which are key for a long-term immunity (immunological memory) [7]. However, the antibody response kinetics in SARS-CoV-2 infection is largely unknown, as well as its clinical value.

Even if serological tests are not as effective as PCR during the acute infection, they can detect antibodies for a long period after disease recovery. Knowledge of a previous infection is epidemiologically crucial and is currently an unmet need in the pandemic.

One of the aims in forefront COVID-19 hospitals, such as the San Paolo University General Hospital in Milan is to protect hospital staff from being infected.

The present study is aimed to evaluate the presence of serum specific antibodies against SARS-CoV-2 by a robust and rapid qualitative test in healthcare workers (HCWs) to explore the possibility of subclinical or asymptomatic infection, and to identify individuals who could have been previously infected.

## Methods

A serological survey was carried out in Milan, Italy, from 2nd April 2020 to 16th April 2020.

A total of 5.7 mL of blood samples were collected from 202 apparently healthy workers of San Paolo University General Hospital. Different types of workers were recruited (Table 1). Peripheral blood was obtained after patient informed consent (“Milano Area 1” Ethical Committee prot. n. 2020/ST/057).

**Table 1:** Descriptive analysis of the cohort recruited in an Italian hospital

*Median*		45 (35–54)
*Age groups, n (%)*	*20–29*	27 (13.4)
	*30–39*	44 (21.8)
	*40–49*	57 (28.2)
	*50–59*	51 (25.3)
	*60–69*	23 (11.4)
*Males, n (%)*		70 (34.7)
*IgG, n (95% CI)*		15; 7.4% (3.8–11.0%)
*IgM, n (95% CI)*		29; 14.4% (9.6–19.2%)
*Swab, n (%)*	*Negative*	22 (10.9)
	*Positive*	7 (3.5)
	*Not done*	173 (85.6)
*Job, n (%)*	*Medical doctors*	95 (47.0)
	*Nurses*	53 (26.2)
	*Medical residents*	20 (9.9)
	*Socio-sanitary worker*	11 (5.5)
	*Administrative staff*	5 (2.5)
	*Technicians*	8 (4.0)
	*Hospital staff*	8 (4.0)
	*Non-hospital staff*	2 (1.0)
*Contact with Covid-19 patients, n (%)*		158 (78.2)
*Median (IQR) temperature, °C*		36.2 (35.8–36.5)
*Normal breathing, n (%)*		202 (100.0)
*Cough, n (%)*		9 (4.5)
*Sore throat, n (%)*		9 (4.5)
*Muscle pain, n (%)*		8 (4.0)
*Malaise, n (%)*		2 (1.0)
*Headache, n (%)*		2 (1.0)
*Anosmia, n (%)*		3 (1.5)
*Dysgeusia, n (%)*		3 (1.5)
*Gastro-intestinal disease, n (%)*		4 (2.0)

The BioMedomics IgM-IgG Combined Antibody Rapid Test (Morrisville, USA), which is a rapid point-of-care lateral flow immunoassays specific for SARS-CoV-2, was adopted for the study. It was validated by the Chinese CDC in the recent past. Its sensitivity and specificity were 88.7 and 90.6%, respectively [8].

### Serological analysis

BioMedomics Rapid IgM-IgG Combined Antibody Test for COVID-19 (IVD-CE certified), immunochromatography based, was used for the present survey. The test card contains colloidal gold-labeled recombinant directed against and quality control antibody colloidal gold marker, two detection lines (G and M), and one quality control line (C) fixed on a nitrocellulose membrane. M and G are fixed with monoclonal anti-human IgM and IgG antibodies for detecting SARS-CoV-2 IgM and IgG antibodies, respectively. The antibody/antigen complex is captured by the anti-human IgM or IgG antibody forming a red M or G line, respectively. If antibodies are missing in the sample, a negative result is showed.

**Fig. 1:**
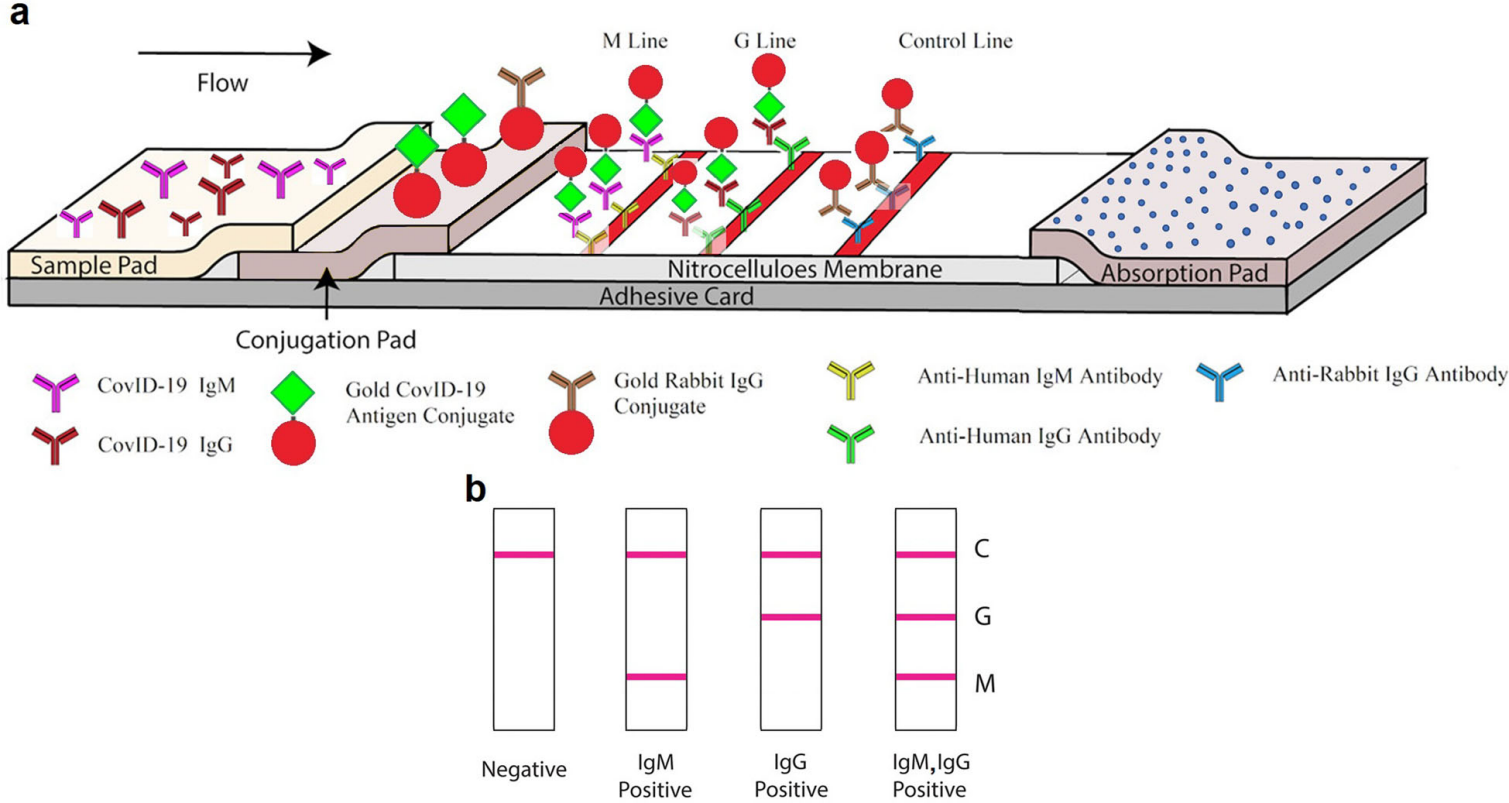
Schematic illustration of rapid SARS-CoV-2 IgM-IgG combined antibody test. **a** Schematic diagram of the detection device; **b** an illustration of different testing results; C, control line; G, means IgG line; M, IgM line. IgG, immunoglobulin G; IgM, immunoglobulin M

Ten μl of serum is added into the sample port followed by the addition of sample dilution buffer. Hydration and transport of reagents are the basis of the assay: they interact with the specimen across the strip via chromatographic lateral flow (Fig. 1a and b).

#### Statistical analysis

An ad hoc electronic form was prepared to collect all study variables. Qualitative variables were summarized with absolute and relative (percentage) frequencies. Quantitative variables were described with medians (interquartile ranges, IQR) for their non-parametric distribution. Prevalence data were provided with point and interval (95% confidence intervals, CI) estimates. Stratified analyses were carried out to assess the prevalence of IgG and IgM in several population subgroups.

Qualitative variables were compared with chi-squared or Fisher exact test, when appropriate.

A two-tailed *p*-value less than 0.05 was considered statistically significant.

All statistical computations were performed with the statistical software Stata version 16 (StataCorp, Texas, USA).

## Results

A total of 202 individuals were retrospectively recruited in the present study (Table 1). The median (IQR) age was 45 (35–54) years (the most represented age classes were 40–49 and 50–59 years with 28.2 and 25.3% of the total sample, respectively). Only 34.7% were male. About half of the cases were medical doctors (95, 47.0%), followed by nurses (53, 26.2%), and medical residents (20, 9.9%).

Median (IQR) body temperature was 36.2 (35.8–36.5)°C, 9 (4.5%) complained of cough, 9 (4.5%) of sore throat, 8 (4.0%) muscle pain, and 3 (1.5%) of anosmia and dysgeusia, respectively.

The prevalence (95% CI) of IgM and IgG positivity was 14.4% (9.6–19.2%) and 7.4% (3.8–11.0%), respectively. The percentage of IgM positivity was statistically higher in males than females (24.3% VS. 9.1%; *p*-value: 0.003), whereas no differences were found in the rate of IgG positivity (10.0% VS. 6.1%; *p*-value: 0.31) (Table 2).

**Table 2:** Seroprevalence of recruited healthcare workers stratified by gender

	Females	Males	***p***-value
IgG*, n (95% CI)*	8/132; 6.1% (2.0–10.2%)	7/70; 10.0% (3.0–17.0%)	0.31
IgM*, n (95% CI)*	12/132; 9.1% (42–14.0%)	17/70; 24.3% (14.3–34.4%)	0.003

Similar immunological differences were found in the age classes: IgM prevalence was higher in the groups 60–69 years and 20–29 years (30.4 and 25.9%, respectively) and lower in the group 40–49 years (5.3%; *p*-value: 0.01) (Table 3). No statistically significant differences were found in the IgG prevalence among the different age groups (*p*-value: 0.33).

**Table 3:** Seroprevalence of recruited healthcare workers stratified by age-group

	20–29 years	30–39 years	40–49 years	50–59 years	60–69 years	***p***-value
*IgG, n (95% CI)*	4/27; 14.8% (1.4–28.2%)	1/44; 2.3% (−2.1–6.7%)	4/57; 7.0% (0.4%13.6%)	5/51; 9.8% (1.6–18.0%)	1/23; 4.4% (−4.0–12.8%)	0.33
*IgM, n (95% CI)*	7/27; 25.9% (9.4–42.4%)	4/44; 9.1% (0.6–17.6%)	3/57; 5.3% (−0.5%; 11.1%)	8/51; 15.7% (5.7–25.7%)	7/23; 30.4 (11.6–49.2%)	0.01

Although the study population worked in the hospital, 78.2% (158) were involved in COVID-19 patients care, the percentage of IgG and IgM positive cases did not differ in case of history of contact with COVID-19 patients in comparison with non-contacts (6.8% VS. 3.5% for IgG, *p*-value: 0.86; 15.9% VS. 13.9% for IgM, *p*-value: 0.74) (Table 4). No information was available on the adherence to the use of personal protective equipment by the healthcare workers. However, recommendations were provided by the local authorities both for suspected patients and healthcare workers.

**Table 4:** Seroprevalence of recruited healthcare workers stratified by exposure to COVID-19 patients

	No-contact	Contact with Covid-19 patients	***p***-value
*IgG, n (95% CI)*	9/44; 6.8% (−0.6–14.2%)	12/158; 7.6% (3.5%; 11.7%)	0.86
*IgM, n (95% CI)*	7/44; 15.9% (5.1–26.7%)	22/158; 13.9% (8.5–26.7%)	0.74

Nasopharyngeal swab was positive and negative in 7 (3.5%) and 22 (10.9%) subjects, respectively (Table 1). The percentage of IgG positivity was higher in individuals with a positive nasopharyngeal swab (57.1% VS. 27.3%; *p*-value: 0.019), whereas IgM prevalence was not significantly higher in individuals with a negative nasopharyngeal swab (95.5% VS. 85.7%; *p*-value: 0.38) (Table 5).

**Table 5:** Seroprevalence of recruited healthcare workers stratified by nasopharyngeal swab positivity

	Negative swab	Positive swab	***p***-value
*IgG, n (95% CI)*	6/22; 27.3% (8.7–45.9%)	4/7; 57.1% (20.4–93.8%)	0.19
*IgM, n (95% CI)*	21/22; 95.5% (86.8–100%)	6/7; 85.7% (59.8–100%)	0.38

A specific hospital job was not associated with an increased proportion of IgG and IgM positivity: medical doctors showed the higher IgG percentage (14.8%), whereas other professional categories than medical doctors and nurses showed the higher IgM prevalence (26.1%) (Table 6). The sero-prevalence of IgG and IgM in non-HCWs and HCWs was not statistically different, even if the few non-HCWs did not show IgG or IgM positivity (Table 7).

**Table 6:** Seroprevalence of recruited healthcare workers stratified by professional activity

	Medical doctors	Nurses/OSS	Others	***p***-value
*IgG, n (95% CI)*	7/115; 14.8% (8.3–21.3%)	5/64; 7.8% (1.2–14.4%)	3/23; 13.0% (−0.7–26.7%)	0.46
*IgM, n (95% CI)*	16/115; 13.9% (7.6–20.2%)	7/64; 10.9 (3.2–18.5%)	6/23; 26.1% (8.1–44.1%)	0.20

**Table 7:** Seroprevalence of recruited healthcare workers stratified by healthcare worker status

	Non-HCWs	HCWs	***p***-value
*IgG, n (95% CI)*	0/5; 0.0%	15/197; 7.6% (4.1–11.7%)	1.00
*IgM, n (95% CI)*	0/5; 0.0%	29/197; 14.7% (9.8–19.6%)	0.38

## Discussion

The present study shows a low IgM prevalence (29, 14.4%) in HCWs working in an Italian hospital with a high burden of COVID-19 patients. The presence of IgM in the serum is potentially associated with an acute phase in the majority of the infections; in particular, the indirect diagnosis based on the assessment of an immunological response against a virus was adopted to ascertain a recent interaction between a virus and the human host. However, the antibody kinetics is complex: patients could not show IgM during the acute infection or, sometimes, can show a combination of IgM and IgG [9]. Then, the timing of the blood collection and the variable early immune response can affect the interpretation of the serological results.

The nasopharyngeal swab, carried out in individuals with serum IgM to confirm the acute SARS-CoV-2 infection, highlighted the limitation of the indirect diagnosis: only 7 cases out of 29 (24.1%) were positive. This can complicate the management of individuals with an IgM positivity: should be they considered contagious and, then, quarantined to avoid the occurrence of an infection with their close contacts? Although the virological tests do not have a diagnostic accuracy of 100% (probability of false positive and negative results), they are the current gold standard for the assessment of the SARS-CoV-2 infection.

A high proportion of the individuals recruited in the present sero-survey (158, 78.2%) stated that they had a contact with COVID-19 patients; however, it was not clearly evaluated the type of contact (e.g., random or close contact, contact duration, etc.). Close contact with contagious patients could be considered a condition associated with the occurrence of the acute infection, but the nasopharyngeal swab was only carried out to individuals with a IgM positivity and/or clinical symptoms suggestive of SARS-CoV-2 infection.

It was found a statistically significant higher prevalence of serum IgM in males than in females (24.3% VS. 9.1%): this issue could highlight a gender imbalance in the infection rate, which was proved by studies where males are at highest risk of infection and severe disease. The high percentage of females in the present study can indirectly support this hypothesis. However, only sero-epidemiological studies performed in the general population could address the present issue. Its clarification could be relevant from a clinical and public health perspective: males could be considered a vulnerable group to protect in case of future epidemic waves.

Another interesting finding is the higher IgM prevalence in individuals in the age groups 20–29 years (25.9%) and 60–69 years (30.4%) in comparison with those aged from 30 to 59 years. This epidemiological imbalance needs to be better evaluated: individuals aged 30–59 years were the majority of the individuals enrolled in the study but the single prevalence of the three age groups was less than 10% in two out of three groups. Adults could be a group biologically more protected or could have been more careful in terms of infection control. However, only ad hoc study designs could solve this important epidemiological question.

A low proportion of medical doctors (14%) shows serum IgM; however, it was the professional category with the high prevalence, although the difference with other categories was not statistically significant.

The IgG prevalence was very low (7.4%) in the recruited sample; it was lower if compared with the IgM prevalence (14.4%). The assessment of the IgG prevalence stratified by several confounding variables (e.g., age, gender, job, contact with COVID-19 patients) does not showcase statistically significant findings. Another important factor which could be associated to the infection rate is the adherence to the recommendations on the use of personal protective equipment (e.g., coveralls, gloves, masks, and goggles). They can reduce the contact with droplets and contaminated surfaces. Guidance on the correct procedure for putting on and removing them was provided in the hospital. However, a direct assessment of the adherence was not carried out.

The antibody prevalence depends on several epidemiological factors; in particular, the exposure to contagious cases (i.e., intensity and/or duration of exposure), the phase of the epidemic (the transition to the interpandemic phase is associated to a higher prevalence if compared to the first epidemic stages), immunological status (e.g., immunocompromised individuals do show a lower prevalence), diagnostic accuracy of the serological test (low sensitivity).

However, the scientific community should address another important issue: the qualitative function of the detected antibodies. It is important to understand if the IgM and IgG antibodies can neutralize the virus, avoiding the infection of susceptible cells.

The present study has several limitations. Although more than 200 individuals recruited in a hospital might have a significant statistical power to show statistically significant differences (e.g., higher prevalence of IgM positivity in males), other studies with a great sample size are recommended.

The present sero-survey is based on a cross-sectional design and, then, cannot prove the incidence of infection and disease in a cohort of patients; the follow-up could be key to better assess the sero-conversion in individuals exposed to contagious patients.

Furthermore, the questionnaire was not tailored to assess the association between independent variables and the occurrence of IgM positivity. The study design and the inclusion of other important confounding variables could help clarify the dynamics of acute infection in some at-risk groups.

Differences found in the present study could not be generalized to the general population in which a very low percentage of positive cases was found [10]. Moreover, the low proportion of some professional categories in this sample cannot help show statistical differences which might be described in the statistical population.

## Conclusions

The findings of our study are key and can help address the role of the serological testing in a population working in a hospital. The results show a low IgM prevalence in more than 200 individuals. Furthermore, a poor correlation between IgM and virological positivity was found thus suggesting that serological testing may be helpful for the diagnosis of suspected patients with negative RT-PCR results and for the identification of asymptomatic infections, as reported by Long et al. [9].

New studies are needed to better explain the role played by the immunological response in the interaction with the human host and to understand the interpretation of the immunological positivity.

## Data Availability

The dataset generated and analyzed during the current study is not publicly available due to an internal policy but is available from the corresponding author on reasonable request.

## Abbreviations

SARS-CoV-2: Severe Acute Respiratory Syndrome Coronavirus 2
RT-PCR: Real time reverse transcriptase polymerase chain reaction
qPCR: Quantitative PCR
HCWs: Healthcare workers
IQR: Interquartile Range
CI: Confidence Interval
